# RELATIONSHIP BETWEEN LEISURE CONSTRAINTS AND WELL-BEING IN MIDDLE TO OLDER ADULTS

**DOI:** 10.1093/geroni/igad104.3562

**Published:** 2023-12-21

**Authors:** Junyan Tian, Angie Sardina, Alexa Allan, Alyssa Gamaldo, Jacqueline Mogle

**Affiliations:** Penn State University, State College, Pennsylvania, United States; UNC Wilmington, Wilmington, North Carolina, United States; The Pennsylvania State University, University Park, Pennsylvania, United States; The Pennsylvania State University, University Park, Pennsylvania, United States; Clemson University, Clemson, South Carolina, United States

## Abstract

Leisure activity engagement is correlated with several health outcomes, such as depression, a lower risk of dementia and better physical health. However, older adults report experiencing leisure constraints in daily lives due to intrapersonal (e.g., stress), interpersonal (e.g., interaction with friends) and structural factors (e.g., availability of opportunity). Since relatively few research investigated the impact of leisure constraints on middle and older adults’ eudaimonic well-being, the major aim of this study was to explore whether perceived leisure constraints are uniquely associated with eudaimonic well-being after adjusting for sociodemographic, loneliness, health and neighborhood covariates. This study accessed leisure constraints using Leisure Barriers and Interest Questionnaire, and accessed eudaimonic well-being by 4 items from the Patient-Reported Outcomes Measurement Information System (PROMIS) questionnaire among 378 adults (age range: 40-92, M = 58.4). Correlation results indicated that better well-being was correlated with less perceived leisure constraints (r = -.40, p < .01), greater household income (r = .14, p = .01), less reported loneliness (r = -.53, p < .01), greater reported neighborhood safety (r = -.18, p < .01), and better reported neighborhood quality (r = .23, p < .01). Linear regressions revealed that individuals who perceived a lower level of leisure constraints associated with better well-being (β = -0.20, p < .01) after adjusting for age, sex, income, health conditions, loneliness, and neighborhood quality and safety. Our results highlight the importance of reducing leisure constraints in intrapersonal, interpersonal and structural levels to help older adults live a meaningful and happy life.

